# Gill Tissue Tolerance Remodeling and Compensatory Regulatory Mechanisms Under Acute Hypoxic Stress in Topmouth Culter (*Culter alburnus*)

**DOI:** 10.3390/antiox15080918

**Published:** 2026-07-24

**Authors:** Jinmei Tang, Huali Zhao, Hao Zhang, Kabba Koroma, Di’an Fang

**Affiliations:** 1 Wuxi Fisheries College, Nanjing Agricultural University, Wuxi 214081, China; 2 Key Laboratory of Freshwater Fisheries and Germplasm Resources Utilization, Ministry of Agriculture and Rural Affairs, Freshwater Fisheries Research Center, Chinese Academy of Fishery Sciences, Wuxi 214081, China

**Keywords:** hypoxic stress, tolerance remodeling, metabolic regulation, oxidative stress

## Abstract

Topmouth culter (*Culter alburnus*) is highly sensitive to hypoxic conditions, but its regulatory mechanisms remain poorly understood. In this study, *C. alburnus* were exposed to hypoxia (DO: 0.60 ± 0.05 mg·L^−1^) for 0, 2, 4, 6, 12, and 24 h to investigate the gill tissue responses and the underlying regulatory mechanisms. Results showed that gill lamellae of *C. alburnus* exhibited distortion and thickening under hypoxic stress for 2–6 h. Between 12 and 24 h of hypoxia, the gill tissue exhibited changes characterized by sinusoidal dilatation and an increased number of red blood cells. Interestingly, the apoptosis rates significantly increased in all experimental groups in response to the hypoxic environment. The plasma glucose (Glu) level increased rapidly at the early stage and then gradually declined. The total protein (TP) level showed a slight elevation. Meanwhile, low-density lipoprotein (LDL) and high-density lipoprotein (HDL) exhibited sustained increases. Hypoxia stress may induce a metabolic transition from early reliance on glucose for energy to later reliance on lipid metabolism. SOD activity gradually increased under hypoxia, while MDA content exhibited an overall upward trend. Oxidative stress-related genes (*foxo1b*, *mapkapk2*, *irs2*, *ppargc1b*) were significantly upregulated to enhance antioxidant capacity during the early phase of hypoxic stress. However, during the late phase of hypoxia, the expression of these genes was significantly downregulated. In conclusion, this study offers a new theoretical basis for gill tolerance remodeling and molecular regulation in *C. alburnus* under acute hypoxic stress.

## 1. Introduction

Topmouth culter (*Culter alburnus*) is an important freshwater fish species for its economic and ecological value. In the past 10 years, *C. alburnus* has been cultivated on a large scale in China, with an output value of nearly 10 billion yuan [1]. The consumer market for *C. alburnus* covers more than 20 countries and regions, including China, the United States, Canada, Japan, South Korea, and the European Union. It occupies a key niche in the pelagic food web of aquatic ecosystems and plays a crucial role in maintaining ecological balance [2]. As a top predator, *C. alburnus* can trigger trophic cascade effects on aquatic communities through its predation pressure [3]. This species exhibits a marked intolerance to hypoxia. However, the regulatory mechanisms underlying hypoxia tolerance are not yet fully elucidated, particularly those involving oxidative stress and antioxidant defense systems [4,5]. Hence, exploring the regulatory characteristics of hypoxia tolerance in *C. alburnus* is of utmost importance for advancing fish domestication and genetic breeding efforts.

Dissolved oxygen (DO) serves as the primary oxygen source for fish and is essential for their metabolic and physiological homeostasis [6]. Hypoxia represents a major environmental stressor for fish and other aquatic organisms [7]. Such events are common in both natural waters and aquaculture systems, driven by global warming, eutrophication, and high stocking densities. A major consequence of hypoxic stress is the disruption of reactive oxygen species (ROS) homeostasis [8]. This leads to increased electron leakage at complexes I and III of the mitochondrial electron transport chain, resulting in significantly elevated production of ROS, such as superoxide (O^2−^) and hydrogen peroxide (H_2_O_2_). When ROS accumulation exceeds the scavenging capacity of the cellular antioxidant system, oxidative stress is triggered [9]. Under normal conditions, ROS are maintained at steady-state levels. However, prolonged hypoxic exposure can overwhelm the antioxidant capacity, resulting in oxidative damage to lipids and proteins. This oxidative imbalance has been implicated in various hypoxia-induced pathologies in fish [10,11]. While previous investigations on *C. alburnus* have primarily examined the effects of dietary supplementation on growth performance and hepatic antioxidant capacity [12], the molecular mechanisms governing its oxidative stress responses under acute hypoxic conditions remain poorly characterized.

The gill is a multifunctional organ that performs not only respiratory gas exchange but also immune defense and environmental sensing, thereby serving as the key tissue in sensing and coping with hypoxic stress. Hypoxia exposure induces structural remodeling of the gill in various fish species [13,14]. In addition, it promotes the activation of cellular processes to preserve tissue homeostasis [15]. For instance, acute hypoxia for 6 h can induce structural changes in gill tissue and enhance cell apoptosis in Japanese flounder (*Paralichthys olivaceus*) [16]. In parallel with these morphological changes, plasma biochemical parameters also undergo dynamic changes. For example, the activities of PDH (pyruvate dehydrogenase) and LDH (lactate dehydrogenase) change as part of the systemic metabolic adaptation to hypoxia [17]. At the molecular level, the antioxidant defense system plays a critical role in mitigating hypoxia-induced oxidative damage. In the gill tissue of *Schizothorax prenanti*, hypoxic stress upregulated the expression of antioxidant enzyme genes (*sod*, *cat*), which reflect an adaptive transcriptional response [18]. Despite these insights from other species, the gill-specific hypoxic stress response and its regulatory mechanisms remain completely unexplored in *C. alburnus*.

Therefore, this study aims to investigate the effects of acute hypoxic stress on gill tissue structure, plasma biochemical parameters, and oxidative stress levels in *C. alburnus*. Compared with other studies, our findings will clarify the specific response patterns in gill tissue of *C. alburnus* at different phases under acute hypoxic stress. The results provided a multi-dimensional dissection of the remodeling and damage processes. These findings will identify a theoretical basis for the sustainable aquaculture of this species and offer insights for future research on tissue repair under hypoxic conditions.

## 2. Materials and Methods

### 2.1. Experimental Animals

Healthy *C. alburnus* [mean initial body weight (IW) = 40.98 ± 0.36 g] were selected as experimental fish from the Jingjiang fish base of the Freshwater Fisheries Research Center. They were one year old and all derived from the self-bred strains maintained at the base. The fish were first acclimated for 15 days in indoor concrete aquaculture tanks and then in indoor recirculating glass aquaria (each with a volume of 400 L) to ensure that they adapted to the final rearing conditions before the experiment. During the acclimation period, the water temperature was maintained at 24 ± 1 °C, pH at 7.8 ± 0.2, and DO at 6.8–7.8 mg·L^−1^. The fish were fed a commercial diet (Tongwei Group, Nanjing, China) twice daily (at 08:00 and 17:00) at a total daily rate of 5% of their body weight. To maintain optimal water quality during acclimation, water was exchanged once every three days, with one-third of the total water volume being replaced each time.

### 2.2. Determination of Critical Threshold for Hypoxia Tolerance

Twenty *C. alburnus* individuals were placed in each of the three recirculating glass aquaria containing aquaculture water. The water was the same as that used during the acclimation period. It was maintained at 24 °C with a DO concentration of 7.5 ± 0.5 mg·L^−1^. Then N_2_ was bubbled into the water to reduce DO levels at a constant rate of 1.0 mg·L^−1^ per hour until reaching 1 mg·L^−1^. A portable DO meter (Hach, HQ40d, LVLD, Loveland, CO, USA) was used to measure water DO and temperature in real time [19]. Three physiological thresholds of *C. alburnus* were determined based on DO concentrations: the hypoxic surfacing point (experimental fish frequently exposed their mouths above the water surface to breathe), the hypoxic coma point (fish lost body balance and showed slow responses), and the asphyxiation point (the point at which 50% of the experimental fish died). Following Tian et al. (2020), the hypoxic surfacing point was taken as the reference for low oxygen stress [20]. In the present study, a slightly higher concentration (0.60 ± 0.05 mg·L^−1^) was chosen to ensure stress induction without causing death.

### 2.3. Acute Hypoxic Stress Experiment and Sample Collection

Fish individuals (*n* = 216) were randomly allocated to three glass aquaria, with 72 fish per tank. The glass aquaria were sealed with plastic film, and a nitrogen bubbling system was used to rapidly adjust the DO concentration to the experimental level (0.60 ± 0.05 mg·L^−1^). During the experiment, DO levels in the water were measured hourly using a portable DO meter, and the stress status of *C. alburnus* was continuously observed. Heating rods were employed to keep the water temperature consistent across all tanks. Experimental fish were anesthetized using MS-222 at a concentration of 100 mg L^−1^. Samples were collected at 0 h (CG), 2 h (2H), 4 h (4H), 6 h (6H), 12 h (12H), and 24 h (24H) after the initiation of hypoxic stress. The 0 h stress group served as the control group. Additionally, 12 fish were randomly selected from each tank at each of the six time points mentioned above. A 1.0 mL syringe was rinsed with a small amount of heparin sodium solution before caudal vein blood collection. From each tank, blood samples were taken from four fish (*n* = 12). The blood samples were centrifuged at 4000 rpm for 10 min at 4 °C to separate the plasma, which was then stored at −20 °C for subsequent analysis of plasma biochemical parameters. After blood collection, the gill tissues were promptly dissected. Gill tissues from two fish per tank were rinsed with phosphate-buffered saline (PBS) to remove residual blood and then fixed in 4% paraformaldehyde. The remaining gill tissues were rapidly frozen in liquid nitrogen and stored at −80 °C (Thermo Scientific Forma 900 Series, Suzhou, China) for long-term storage. All fish procedures were performed in accordance with the Regulations for the Administration of Animal Laboratory Affairs.

### 2.4. H&E Staining

Hematoxylin-eosin (H&E) staining was performed according to the literature report. After rinsing with PBS, the samples were dehydrated through a graded concentration of ethanol. Then the tissues were incubated in xylene. Subsequently, the samples were embedded in low-melting-point paraffin (56–58 °C) and sectioned into 4 μm thick slices using a microtome. After dewaxing and H&E staining, the sections were examined under a light microscope (WALINOVA, CM2000, Beijing, China) for imaging (*n* = 3). The magnification was 20×.

### 2.5. TUNEL Staining

After tissue sections were prepared as described above, they were treated with proteinase K working solution and incubated at 37 °C for 22 min. Subsequently, the membrane permeabilization solution was added dropwise, and the sections were incubated at room temperature for 20 min. After each incubation step, the sections were washed with PBS three times for 5 min each time. Terminal deoxynucleotidyl transferase dUTP nick end labeling (TUNEL) staining was performed following the manufacturer’s instructions for the TUNEL kit (Servicebio, Wuhan, China). After counterstaining cell nuclei with 4′, 6-diamidino-2-phenylindole (DAPI), the sections were mounted with coverslips, and then photographed under a microscope. ImageJ software (v 1.52a) was employed to quantify apoptotic cells (green fluorescence) and total cells (blue fluorescence) following TUNEL staining, to determine the cell apoptosis rate. One random microscopic field per section was selected for counting. A total of three microscopic fields were examined per group (*n* = 3). TUNEL positive cell ratio = Number of apoptotic cells/Total number of cells × 100%.

### 2.6. Determination of Plasma Biochemical Indices

To assess the intensity of stress response induced by hypoxic stress, the plasma levels of the following biochemical indices were measured: low-density lipoprotein (LDL), high-density lipoprotein (HDL), aspartate transaminase (AST), lactate dehydrogenase (LDH), glucose (Glu), total cholesterol (TC), triglycerides (TG), total protein (TP), albumin (ALB), and alkaline phosphatase (ALP). All detection kits were purchased from Mindray Medical International Co., Ltd. (Shenzhen, China). Analyses were performed using an automatic biochemical analyzer (Model: BS-400 Q2080; Mindray, Shenzhen, China).

### 2.7. Determination of Antioxidant Enzyme Activities and Gene Expression

#### 2.7.1. Antioxidant Enzyme Activity Assays

Gill samples were homogenized with sterile cold physiological saline at a ratio of 1:9 [mass (g):volume (mL)]. After centrifugation at 4 °C in a refrigerated centrifuge for 10 min (5000 r/min), the supernatant was collected to measure the activities of SOD and the MDA content. All experimental procedures were performed according to the corresponding commercial detection kits (Nanjing Jiancheng Bioengineering Institute, Nanjing, China).

#### 2.7.2. Real-Time Polymerase Chain Reaction (PCR) Analysis

Total RNA was isolated from the gill tissues of *C. alburnus* using Trizol reagent combined with isopropanol precipitation. RNA integrity was evaluated via 1% agarose gel electrophoresis, whereas its purity and concentration were quantified using a spectrophotometer. High-quality RNA was reverse-transcribed into cDNA using the PrimeScript^TM^ RT Reagent Kit with gDNA Eraser (Perfect Real Time) (Takara, Dalian, China). Subsequently, qRT-PCR was conducted according to the instructions of the TB Green^®^ Premix Ex TaqTM II Kit (Takara, Kyoto, Japan), using *β-actin* as the reference gene [21]. The qRT-PCR program was listed as follows: initial denaturation at 95 °C for 30 s; then 40 cycles of denaturation at 95 °C for 5 s and annealing/extension at 60 °C for 30 s. Based on the cycle threshold (Ct) values obtained from RT-qPCR, the relative expression levels of oxidative stress-related genes in gill tissues were calculated using the 2^−∆∆Ct^ method [22]. All primers were synthesized by Sangon Biotech (Shanghai, China) Co., Ltd. (Table 1).

### 2.8. Data Processing and Statistical Analysis

SPSS 25.0 was used for the statistical analysis of experimental data. All data were tested using the Shapiro–Wilk test to confirm compliance with the normal distribution, and simultaneously met the homogeneity of variances requirement verified by Levene’s test. For differential analysis among all groups, Duncan’s multiple range test was used following the one-way analysis of variance. All analysis results were expressed as mean ± standard error (mean ± S.E.).

## 3. Results

### 3.1. Critical Hypoxia Tolerance Thresholds of C. alburnus

Preliminary experiments showed that at a water temperature of 24 °C, the hypoxic surfacing point, hypoxic coma point, and asphyxiation point of *C. alburnus* were 0.55 mg·L^−1^, 0.46 mg·L^−1^, and 0.38 mg·L^−1^, respectively. Given that DO levels below the aforementioned critical hypoxic thresholds may adversely affect the aquaculture of *C. alburnus* in practical farming settings, this study focused on the responses near the hypoxic surfacing point. The experimental concentration for hypoxic stress was set at 0.60 ± 0.05 mg·L^−1^.

### 3.2. Gill Tissue Remodeling Under Hypoxic Stress

#### 3.2.1. Morphological Alterations of Gills to Hypoxic Stress

**Figure 1:**
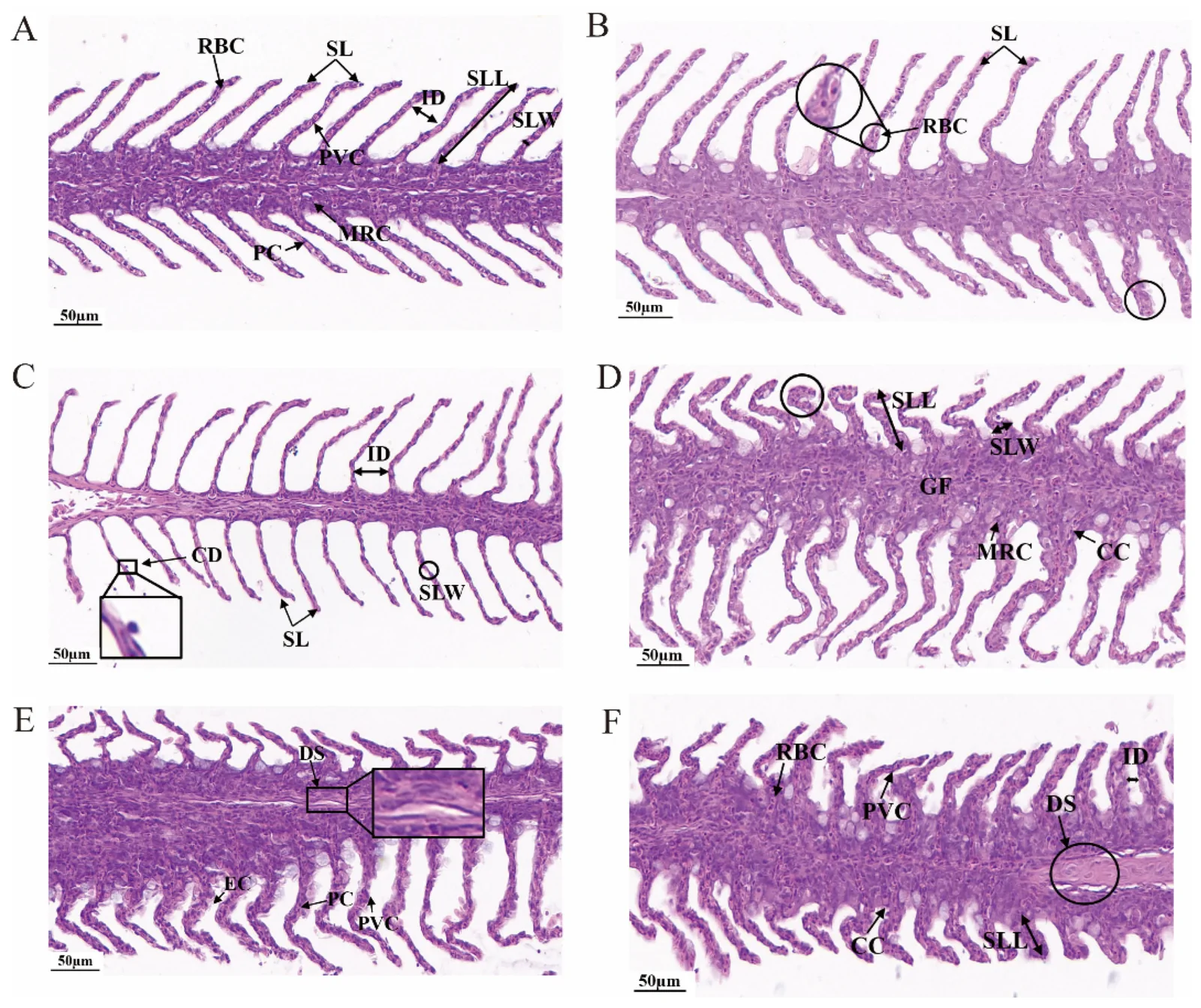
Effect of acute hypoxic stress on gill tissue structure of *C. alburnus*. (**A**) The control group. (**B**) Acute hypoxia 2H group. (**C**) Acute hypoxia 4H group. (**D**) Acute hypoxia 6H group. (**E**) Acute hypoxia 12H group. (**F**) Acute hypoxia 24H group. SLL: secondary lamellar length; SLW: secondary lamellar width; ID: interlamellar distance; GF: Gill filament; SL: Gill lamella; RBC: red blood cell; PC: Pillar cell; PVC: Squamous epithelial cell; MRC: Mitochondria-rich cell; CD: Epithelial cell shedding; CC: Chloride cell; EC: Epithelial cell; DS: Dilation of the blood sinus. Circles in (**B**,**D**) indicate the distal ends of gill lamellae with swelling and thickening. The scale bar is 50 μm.

Gills serve as the primary site for gas exchange in fish, and are highly sensitive in their structural responses to acute hypoxic stress. Morphological observations of the gill tissues were performed via H&E staining. Under normal conditions, the gill filaments of *C. alburnus* were intact and slender; red blood cells (RBCs) exhibited a biconcave disc shape, squamous epithelial cells (PVCs) resembled thin scales, and oval mitochondria-rich cells (MRCs) were observed at the base of the gill lamellae (Figure 1A). After 2 h of hypoxic stress, the gill tissue began to exhibit initial remodeling, characterized by slight twisting of gill lamellae and partial swelling of their terminal regions (Figure 1B). After 4 h of hypoxic stress, the gill lamellae exhibited reduced width and increased interlamellar spacing, accompanied by minor detachment of epithelial cells (Figure 1C). After 6 h of hypoxic stress, compensatory and injurious changes coexisted in the gill tissues of *C. alburnus*: the gill lamellae exhibited shortening, thickening, and increased curvature; the sinusoids widened; the stroma showed slight protrusion; and the numbers of MRCs and Chloride cells (CCs) were increased (Figure 1D). After 12 h of hypoxic stress, the sinusoids in the gill filaments further expanded; the bases of the gill lamellae fused with the stroma; cellular arrangement was disorganized; and local epithelial cell hyperplasia was observed (Figure 1E). Upon extending the hypoxic stress duration to 24 h, the gill lamellae displayed a marked reduction in length; the stroma protruded and fused with the gill lamellae; sinusoids were extensively widened; erythrocytes increased in both number and size; widespread hyperplasia of PVCs was observed; and the number of CCs was substantially reduced (Figure 1F). Collectively, with the prolongation of hypoxic stress, the gill remodeling process gradually intensified, resulting in impairment of both gill structural integrity and osmoregulatory function.

#### 3.2.2. Temporal Apoptosis in Gills

**Figure 2:**
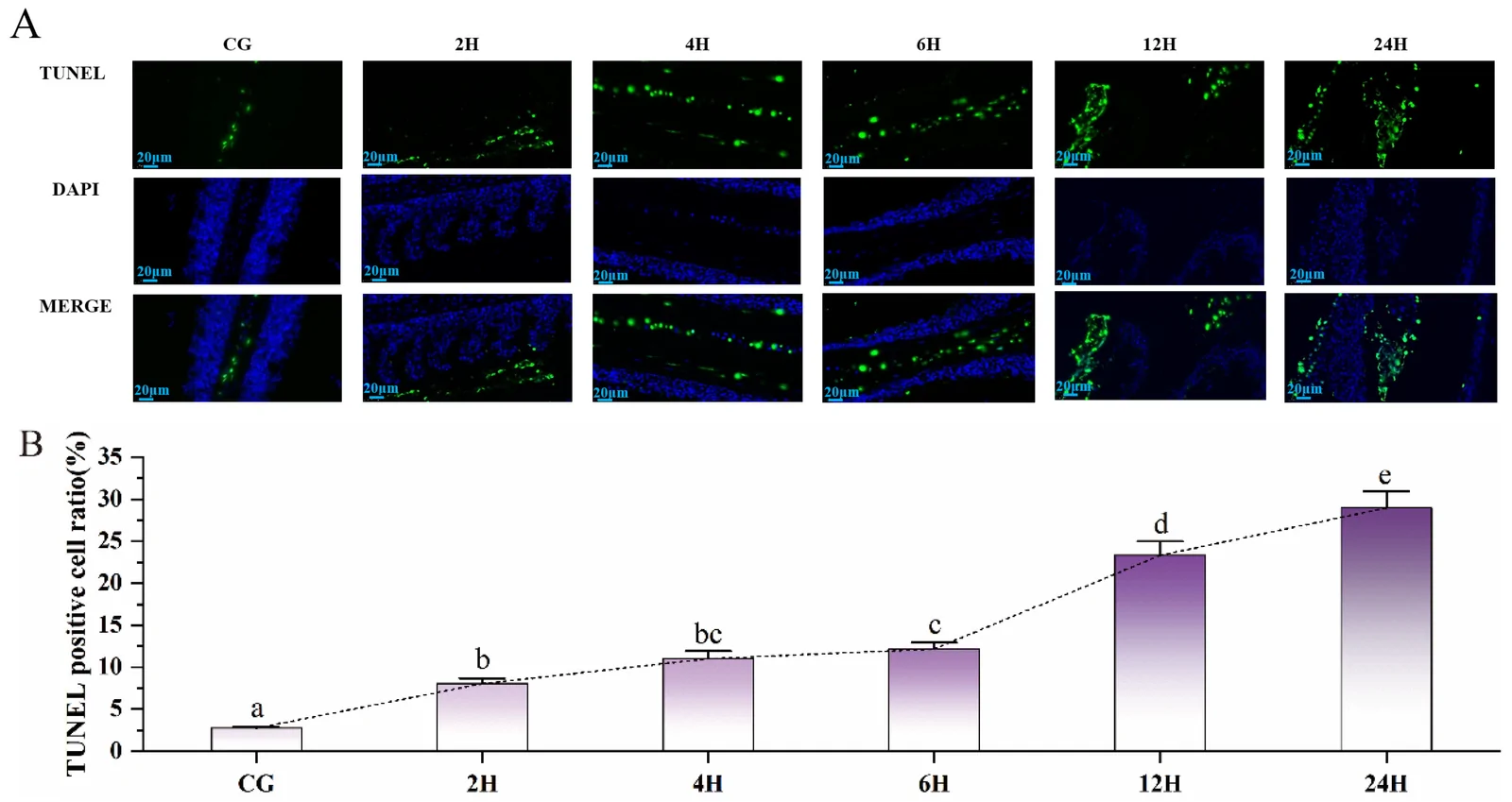
Effect of acute hypoxic stress on apoptosis in the gill tissue of *C. alburnus*. CG: The control group; 2H: Acute hypoxia 2H group; 4H: Acute hypoxia 4H group; 6H: Acute hypoxia 6H group; 12H: Acute hypoxia 12H group; 24H: Acute hypoxia 24H group. (**A**) Under a fluorescence microscope, apoptotic cells show green fluorescence, and nuclei show blue color. The scale bar is 20 μm. (**B**) Variation trend of TUNEL positive cell rate in *C. alburnus*. In the figure, different letters indicate significant differences between the corresponding data groups, while the same letters represent no statistically significant differences. Values with different letters are all statistically significant (*p* < 0.05). The bars represent mean values, and the error bars are standard error (*n* = 3).

TUNEL staining was conducted on the gill tissues of *C. alburnus* to detect apoptotic cells. Results showed that most gill cell nuclei in the CG group remained intact, whereas the gill tissues of hypoxic stress groups exhibited distinct apoptotic signals (Figure 2A). Figure 2B illustrates the apoptosis rates of gill cells following different durations of hypoxic stress. Notably, compared with the control group, the apoptosis rates in the 2H, 4H, 6H, 12H, and 24H hypoxic stress groups were all significantly higher (*p* < 0.05), with an upward trend (Figure 2B).

### 3.3. Temporal Analysis of Physiological Responses Under Hypoxic Stress

**Figure 3:**
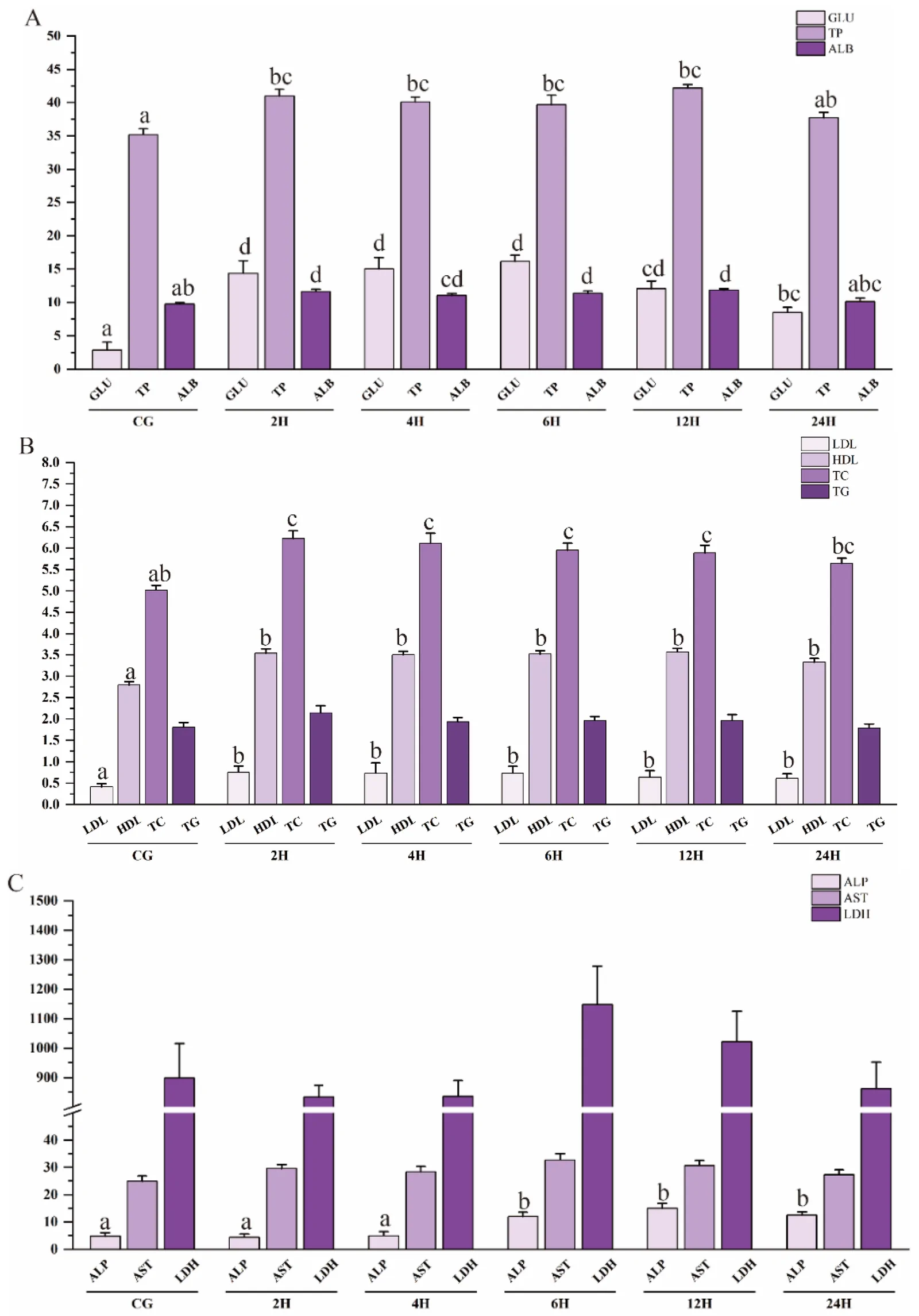
Changes in plasma biochemical indexes of *C. alburnus* under acute hypoxic stress. CG: The control group; 2H: Acute hypoxia 2H group; 4H: Acute hypoxia 4H group; 6H: Acute hypoxia 6H group; 12H: Acute hypoxia 12H group; 24H: Acute hypoxia 24H group. (**A**) Variations in plasma carbohydrate metabolism and protein-related indices of *C. alburnus* under acute hypoxic stress. Glu: Glucose (mmol/L); TP: Total protein (g/L); ALB: Albumin (g/L). (**B**) Variations in plasma lipid metabolism-related indices of *C. alburnus* under acute hypoxic stress. LDL: Low-density lipoprotein (mmol/L); HDL: High-density lipoprotein (mmol/L); TC: Total cholesterol (mmol/L); TG: Triglycerides (mmol/L). (**C**) Variations in plasma metabolic enzyme activities of *C. alburnus* under acute hypoxic stress. ALP: Alkaline phosphatase (U/L); AST: Aspartate transaminase (U/L); LDH: Lactate dehydrogenase (U/L). In the figure, different letters indicate significant differences between the corresponding data groups, while the same letters represent no statistically significant differences. Values with different letters are all statistically significant (*p* < 0.05). The bars represent mean values, and the error bars are standard error (*n* = 12).

The glucose and lipid profile exhibited prominent responses to hypoxic stress. Compared with the CG group, the Glu content increased significantly at the early stage of stress exposure (*p* < 0.05), exhibiting a typical pattern of stress-induced hyperglycemia. It was still significantly higher than that in the CG group after 24 h (*p* < 0.05). The TP content showed minor fluctuations during the period of hypoxic stress and was significantly elevated at 2 h, 4 h, 6 h, and 12 h compared with the CG group (*p* < 0.05). The ALB content showed a bimodal pattern at 2 h and 12 h and was also significantly elevated at 2 h, 4 h, 6 h, and 12 h compared with the CG group (*p* < 0.05) (Figure 3A). The LDL content in plasma was significantly increased (*p* < 0.05), accumulating continuously from 2 h to 6 h and then decreasing at 12 h and 24 h. The HDL content in the CG group was significantly lower than that in all hypoxic experimental groups (*p* < 0.05) and exhibited a continuous and stable increase within 24 h. TC and TG also exhibited fluctuations. Specifically, the TC content was significantly increased from 2 h to 12 h of hypoxia (*p* < 0.05). The TG content showed no statistically significant difference but exhibited an overall upward trend (*p* > 0.05) (Figure 3B). In terms of tissue damage and function assessment, the ALP activity in the 6 h, 12 h, and 24 h hypoxic stress groups was significantly higher than that in the CG group and the 2 h and 4 h hypoxic stress groups (*p* < 0.05), reaching the peak at 12 h, followed by a decrease. The activities of AST and LDH did not change significantly at the early stage of stress (*p* > 0.05). However, LDH activity showed an upward trend after 6 h of hypoxia (Figure 3C).

### 3.4. Effects on Antioxidant Enzyme Activities and Related Gene Expression

**Figure 4:**
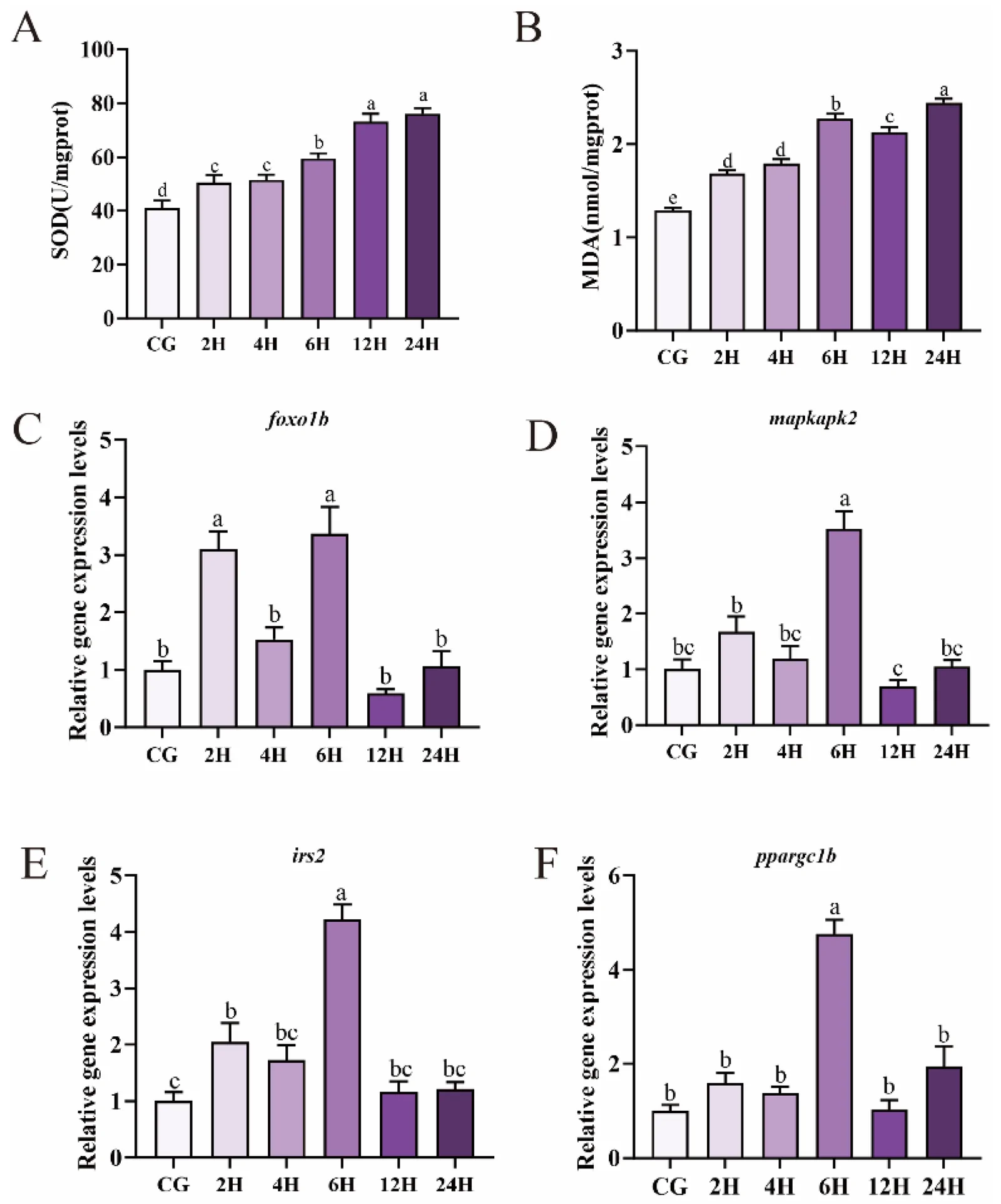
Effects of acute hypoxic stress on antioxidant enzyme activities and related gene expression in the gill tissue of *C. alburnus*. CG: The control group; 2H: Acute hypoxia 2H group; 4H: Acute hypoxia 4H group; 6H: Acute hypoxia 6H group; 12H: Acute hypoxia 12H group; 24H: Acute hypoxia 24H group. (**A**) SOD, superoxide dismutase (U/mgprot). (**B**) MDA, malondialdehyde (nmol/mgprot). (**C**) *foxo1b*: Forkhead box transcription factor O1b. (**D**) *mapkapk2*: Mitogen-activated protein kinase activator protein kinase 2. (**E**) *irs2*: Insulin receptor substrate 2. (**F**) *ppargc1b*: Peroxisome proliferator-activated receptor gamma coactivator 1β. In the figure, different letters indicate significant differences between the corresponding data groups, while the same letters represent no statistically significant differences. Values with different letters are all statistically significant (*p* < 0.05). The bars represent mean values, and the error bars are standard error (*n* = 6).

This study examined temporal changes in antioxidant enzyme activities and expression levels of oxidative stress-related genes. The results showed that SOD activity exhibited a gradually increasing trend after hypoxia exposure, and all hypoxia groups were significantly higher than the CG group (*p* < 0.05). No significant difference was observed between the 2H and 4H groups, nor between the 12H and 24H groups (*p* > 0.05) (Figure 4A). MDA content began to increase significantly after 2 h of hypoxia (*p* < 0.05). It showed a transient decrease at 12 h, followed by a subsequent rise, and reached its peak in the 24H group. Overall, MDA content exhibited an increasing trend (Figure 4B).

Additionally, the results showed that the relative expression of *foxo1b* exhibited a biphasic response, with two peaks at 2 h and 6 h of hypoxia, both significantly higher than those in the control group (*p* < 0.05), followed by a decrease to the lowest level at 12 h and a rebound at 24 h (Figure 4C). The mRNA expression of *mapkapk2* peaked only at 6 h of stress (*p* < 0.05), showed a modest increase at 2 h, and gradually recovered after declining at 12 h (Figure 4D). Furthermore, the relative expression level of *irs2* was significantly upregulated at 2 h of hypoxia (*p* < 0.05). It slightly decreased at 4 h of hypoxia, then markedly increased again at 6 h of hypoxia (*p* < 0.05). During the 12–24 h hypoxic period, the expression level decreased and showed no significant difference compared with the CG group (*p* > 0.05) (Figure 4E). The mRNA expression of *ppargc1b* showed no significant change during 2–4 h of hypoxia and reached the highest expression at 6 h. It returned to the CG group level after 12 h of hypoxia, and then increased slightly at 24 h (*p* > 0.05) (Figure 4F).

## 4. Discussion

### 4.1. Structural Remodeling of Gill Tissue in Response to Hypoxic Stress

The gill is a core organ for fish respiratory metabolism, ion regulation, and response to environmental stress. Its structural integrity directly affects the fish’s environmental adaptability, and can also serve as a sensitive biomarker for assessing evaluating ecological and the toxicity of waterborne pollutants [23,24]. Among its components, gill lamellae serve as the key structural basis for gas exchange [25], while CC and MRC play important roles in ion balance and energy metabolism [26]. Prior to hypoxic stress, the gill tissue remained structurally intact. Following exposure to hypoxic stress for 2–6 h, the numbers of MRCs and CCs were increased. Mild distortion and terminal enlargement of the gill lamellae were also observed. These may represent an active adaptive strategy of *C. alburnus* to improve oxygen uptake efficiency. Together with the slight initial upregulation of the cell apoptosis rate (Figure 2B), this could represent a regulatory mechanism to eliminate damaged cells and maintain the functional homeostasis of the gill tissue. It is speculated that apoptosis is induced by excessive ROS in order to maintain the homeostasis of organisms [27]. These two processes together enhance the adaptability to hypoxic environments. This result is relatively similar to the findings in the grass carp (*Ctenopharyngodon idella*), which alleviates hypoxic damage through remodeling of gill lamellae via elongation of mitochondrial cristae [28,29]. During this stage, HDL levels increased steadily (Figure 3B), which compensated for the energy deficit in aerobic metabolism under hypoxia and provided sustained energy for the compensatory process. When hypoxic stress persisted for 12–24 h, significant contraction and stromal fusion of gill lamellae, abnormal dilation of sinusoids, and epithelial cell hyperplasia were observed. This pattern is consistent with the gill tissue remodeling of crucian carp (*Carassius auratus*) under long-term hypoxia [30]. This may be an adaptive strategy of *C. alburnus* to increase the surface area for gas exchange and achieve efficient ion transport.

### 4.2. Physiological Response Mechanism

When dissolved oxygen in water fails to meet the metabolic demands of fish, blood biochemical indicators reflecting fish physiological functions undergo corresponding changes. Changes in these indicators are closely correlated with the intensity of hypoxic stress. Glu serves as a core energy metabolism marker for fish in response to hypoxic stress, which can directly reflect glycolytic activity and the body’s energy compensation capacity [31]. Fluctuations in the concentrations of LDL and HDL are closely related to lipid metabolism regulation; ALP is involved in tissue repair, energy metabolism, and membrane function maintenance. Plasma TG content is commonly used to evaluate lipid catabolism levels [32], while plasma TP content can indirectly reflect the nonspecific immune status of fish [33].

In the early stage of hypoxic stress, the plasma Glu content of *C. alburnus* showed an upward trend, indicating that gluconeogenesis and glycogenolysis pathways were rapidly activated under acute hypoxia. Metabolomic analysis of small yellow croaker (*Larimichthys polyactis*) similarly showed increased gluconeogenesis at the early phase of hypoxic stress [34]. However, Glu content decreased after 6 h. This decrease may be due to the initiation of negative feedback regulation or the depletion of glycogen reserves. This change pattern is consistent with the research results of American paddlefish (*Polyodon spathula*) [35]. It is further speculated that the plasma Glu content in *C. alburnus* may continue to decrease during post-hypoxia reoxygenation [36,37]. During hypoxic stress, the TP and ALB contents in *C. alburnus* increased significantly, which is consistent with the report of increased ALB content in triploid rainbow trout (*Oncorhynchus mykiss*). The increase in these indicators may have a positive impact on the immune function of fish and help alleviate hypoxic stress damage [38,39]. The increase in LDL and HDL levels implies that they may provide alternative energy substrates for the body and reduce oxidative damage through reverse cholesterol transport (Figure 3B). This is consistent with reports documenting elevated HDL levels in turbot (*Scophthalmus maximus*) exposed to hypoxic conditions [40]. No significant fluctuations were observed in TC and TG levels. This is similar to findings that acute hypoxic stress does not exert a notable impact on TG concentrations in Nile tilapia (*Oreochromis niloticus*) [41]. ALP activity remained relatively elevated during the late phase of hypoxic stress (Figure 3C). This pattern is distinct from that observed in other fish species, where ALP activity typically rises initially and then declines under hypoxic exposure [42,43]. This unique response suggests that *C. alburnus* has a restricted capacity to tolerate prolonged hypoxia. Overall, LDH activity showed no statistically significant change (Figure 3C), which matches observations in mummichog (*Fundulus heteroclitus*) subjected to hypoxic stress [44]. However, a subtle increase in LDH activity was detected after 6 h of hypoxia exposure. It may indicate a transient upregulation of anaerobic metabolic processes that did not result in severe cellular damage. The adaptive morphological alterations in the gill lamellae were also observed under this metabolic pattern. In summary, *C. alburnus* exhibited a shift from aerobic to anaerobic metabolism and targeted adjustments in lipid pathways under hypoxic stress.

### 4.3. Hypoxia Adaptation and Oxidative Stress Responses

Numerous studies have shown that fish experience oxidative stress responses under hypoxic stress [45]. When oxidative stress occurs, ROS accumulate in large amounts in the fish body. Once the level of ROS exceeds the capacity of the antioxidant system, lipid peroxidation occurs. MDA is a secondary product of lipid peroxidation and serves as a marker of oxidative stress [46]. Antioxidant enzymes, including SOD, are the first line of defense to neutralize or scavenge ROS [47]. This study found that with the extension of hypoxic time, SOD activity showed an increasing trend. This has also been observed in common carp [48]. It is speculated that this is an adaptive defense response initiated to scavenge excessive free radicals. MDA content gradually increased during 2–6 h of hypoxia, then briefly decreased at 12 h. It increased again and reached its peak at 24 h. The continuous increase in MDA suggests that the antioxidant system is impaired and oxidative damage is aggravated. The same finding has been observed in *O. mykiss* [22].

Gill tissue remodeling is a core adaptive strategy of *C. alburnus* in response to hypoxic stress. The synergistic regulation of oxidative stress-related genes plays a key role in this process. This study focused on four oxidative stress-related genes, *foxo1b*, *mapkapk2*, *irs2*, and *ppargc1b*. It was found that their expression levels showed an increasing trend during the early phase of hypoxic stress. As a core transcription factor of the FoxO signaling pathway, *foxo1b* is widely involved in the hypoxic stress response [49]. Additionally, *foxo1b* engages in the formation of a transcriptional regulatory complex with *ppargc1b* to govern mitochondrial biogenesis [50]. Activation of *foxo1b* promotes mitophagy, which may in turn facilitate the release of pro-apoptotic factors [51]. This might thereby explain the significant increase in apoptotic cells. The concomitant upregulation of *mapkapk2* may preserve the integrity of gill epithelial cells under hypoxic conditions via cytoskeletal stabilization [52]. This laid the structural foundation for the terminal enlargement of gill lamellae. The mRNA expression of *irs2* exerts negative feedback regulation on *foxo1b* through the PI3K-AKT signaling pathway [53]. The upregulation of *irs2* prevented excessive activation of the antioxidant response. From 12 h to 24 h of hypoxic stress, the expression levels of *foxo1b*, *mapkapk2*, *irs2*, and *ppargc1b* were significantly down-regulated, presumably because the accumulation of oxidative stress and inflammatory factors exceeded the tolerance threshold of the organism. Combined with the sustained increase in MDA content (Figure 4B), these results indicated that hypoxic stress caused severe damage to the gill tissue of *C. alburnus*. These four oxidative stress-related genes exhibited a stage-specific fluctuating expression pattern of initial increase followed by a decrease (Figure 4), which is consistent with the hypoxic response pattern in the muscle tissue of *O. mykiss* [22]. This pattern is speculated to represent an adaptive response of the gill tissue of *C. alburnus* to hypoxic environments [54]. Strikingly, the 12 h time point represented a conserved turning event. It defined a critical threshold that signified the progression of the antioxidant system from adaptive homeostatic adjustment to degenerative damage.

## 5. Conclusions

This study systematically characterized the stage-specific features and regulatory mechanisms governing gill tissue remodeling in *C. alburnus* exposed to hypoxic stress. Transcriptional activation of oxidative stress-related genes along with significantly elevated antioxidant enzyme activities secured the antioxidant defense function. And these changes together with metabolic reprogramming supported adaptive remodeling of the gills. With the persistence of hypoxia, antioxidant capacity declined, accompanied by exacerbated apoptosis. This ultimately drove the shift in gill remodeling from adaptive adjustment to pathological deterioration. Summarily, this study investigated the gill remodeling process of *C. alburnus* under hypoxic stress, providing new insights into the theoretical basis of oxidative stress in regulating hypoxic adaptation in fish.

## Figures and Tables

**Table 1:** Primer sequences for oxidative stress-related genes.

Gene Name	Primer Sequence, (5′ to 3′)	Amplicon Lengths	GenBank Accession Number
*foxo1b*	F: ATCCCATTCGCCTCGGTAAC R: GTTGCCGTTGGTGTAACTCG	107 bp	KAK9968397.1
*mapkapk2*	F: AAGACTCGTCCAATCCGCTG R: TTAGCAAGCAGGCGTCTCAT	83 bp	KAK9966504.1
*irs2*	F: TGAGGAACAGCGAAGGATCG R: TGAACCGGACAAGCGAATCA	144 bp	KAK9969665.1
*ppargc1b*	F: TTCGGCAGGAAGCGTTACAT R: GCGTCAAAGTCCAGTGCATC	83 bp	KAK9962950.1
*β-actin*	F: ACTTCGAGCAGGAGAT R: ACAGTGTTGGCATACAG	229 bp	GU217817.1

## Data Availability

The original contributions presented in this study are included in the article. Further inquiries can be directed to the corresponding author.

## Abbreviations

The following abbreviations are used in this manuscript:

DO	dissolved oxygen
ROS	reactive oxygen species
IW	mean initial body weight
PBS	phosphate-buffered saline
LDL	low-density lipoprotein
HDL	high-density lipoprotein
AST	aspartate transaminase
LDH	lactate dehydrogenase
Glu	glucose
TC	total cholesterol
TG	triglycerides
TP	total protein
ALB	albumin
ALP	alkaline phosphatase
HE	hematoxylin-eosin
TUNEL	terminal deoxynucleotidyl transferase-mediated dUTP nick end labeling
RT-qPCR	real-time polymerase chain reaction
Ct	cycle threshold
GF	gill filament
SL	gill lamella
BC	blood cell
PC	pillar cell
PVC	squamous epithelial cell
MRC	mitochondria-rich cell
CD	epithelial cell shedding
CC	chloride cell
EC	epithelial cell
DS	dilation of the blood sinus
